# Perceived Social Isolation and Conspiracy Mentality: Exploring Pathways to Anti‐LGBTQ+ Conspiracy Beliefs

**DOI:** 10.1111/sjop.70111

**Published:** 2026-04-29

**Authors:** Sara Panerati, Barbara Barcaccia, Muslumogullari Yunus Emre, Marco Salvati

**Affiliations:** ^1^ Department of Human Sciences University of Verona Verona Italy; ^2^ Department of Education Roma Tre University Rome Italy; ^3^ Associazione di Psicologia Cognitiva APC and Scuola di Psicoterapia Cognitiva SPC Rome Italy

**Keywords:** anti‐LGBTQ+ conspiracy beliefs, conspiracy mentality, mediation model, social isolation

## Abstract

Conspiracy beliefs have long been a recurring feature of human society; however, when they target disadvantaged groups, such as the LGBTQ+ people, they represent a particularly harmful phenomenon with detrimental consequences. Despite the increasing interest in the phenomenon, research to date has often overlooked the influence of an individual social environment. Indeed, recent evidence highlighted that individuals who experience limited social integration might be more vulnerable to adopting a conspiratorial mentality. In this regard, social isolation may represent a possible trigger, fuelling epistemic uncertainty and existential anxiety, and enhancing susceptibility to anti‐LGBTQ+ conspiracy beliefs. Hence, the current study (*N* = 820) investigated whether individuals who experience heightened levels of perceived social isolation are more likely to report high levels of anti‐LGBTQ+ conspiracy beliefs endorsement via an enhanced conspiratorial mentality. Results were in line with our expectations, pointing out positive associations between perceived social isolation and conspiracy beliefs, both directly and indirectly, as a function of greater levels of conspiracy mentality. Therefore, an individual's social isolation may represent a promising approach to highlighting the factors that produce fertile ground for the endorsement of anti‐LGBTQ+ conspiracy beliefs and may represent a promising aspect for identifying potential pathways for intervention and prevention.

## Keypoints

- The present research examines the associations between perceived social isolation and the endorsement of conspiracy beliefs targeting LGBTQ+ people by focusing on conspiracy mentality as a general dispositional orientation that is conceptually distinct from, yet systematically related to, specific conspiracy beliefs.
- Findings showed that participants reporting a higher level of perceived social isolation were more likely to report a greater endorsement of anti‐LGBTQ+ conspiracy beliefs.
- The results showed an indirect association via the conspiracy mentality, indicating that higher levels of perceived social isolation were associated with higher levels of conspiracy mentality, which, in turn, were related to a higher endorsement of anti‐LGBTQ+ conspiracy beliefs.
- The individual's social context may represent a useful approach to highlight the factors that produce fertile ground for the endorsement of conspiracy beliefs.

Conspiracy beliefs have long been a recurring feature of human societies and have historically been used to make sense of complex and threatening realities (Hornsey et al. 2023; van Prooijen and van Vugt 2018). Rather than being a novel phenomenon, conspiracy beliefs become particularly socially consequential when they are mobilized to legitimize social exclusion, discrimination, and the erosion of fundamental rights (Bayar 2025). When conspiracy beliefs target marginalized social groups, such as LGBTQ+ people, their societal impact may be further amplified, contributing to stigma, social exclusion, and opposition to policies toward equality. In this regard, recent international reports documented a persistent backlash against LGBTQ+ rights across several countries, including increased political mobilization against gender diversity, restrictions on legal protections, and the spread of hostile hate speech toward sexual and gender minorities (European Commission 2020; ILGA‐Europe 2024). Therefore, it is essential to grasp the social conditions that nurture these beliefs, as they can indirectly fuel ongoing discrimination and the erosion of support for LGBTQ+ rights.

Despite the increasing interest in understanding the antecedents of the phenomenon, research to date has predominantly focused on investigating either individual characteristics associated with the endorsement of conspiracy beliefs (e.g., social dominance orientation; Salvati et al. 2022) or their behavioral and attitudinal consequences (e.g., discriminatory attitudes, refraining from voting; Jolley et al. 2023). Hence, despite the useful insights recent studies provide, they usually do not take into account the individual social environment (Phadke et al. 2021).

In this regard, recent evidence has highlighted that individuals with limited social integration or who lack a sense of belonging may be more vulnerable to displaying a conspiratorial mentality (i.e., an underlying disposition that promotes the adoption of conspiracy beliefs; Schnell et al. 2024) and that loneliness and social disconnection are systematically associated with stronger endorsement of conspiracy theories (Bierwiaczonek et al. 2024).

From a theoretical perspective, this vulnerability can be framed within the Existential Threat Model (ETM; van Prooijen 2020), which posits that experiences that undermine an individual's fundamental needs (i.e., security, belonging, and meaning) can constitute existential threats that promote conspiratorial thinking. In this regard, social isolation constitutes a condition of heightened existential insecurity that might be associated with increased individuals' reliance on conspiratorial worldviews as a compensatory meaning‐making strategy. Consistent with this reasoning, empirical evidence shows that (experience of) social exclusion and social threat are associated with increased endorsement of conspiracy theories (Graeupner and Coman 2017; Poon et al. 2020). In this regard, social isolation can fuel epistemic uncertainty and existential anxiety (Miceli and Castelfranchi 2005), thereby increasing the appeal of conspiratorial narratives that offer simplistic explanations for complex social realities.

Within this framework, conspiracy beliefs targeting disadvantaged social groups, such as the LGBTQ+ community, represent a particularly harmful phenomenon (Mouafo 2023), portraying sexual and gender minorities as a coordinated and “powerful lobby” allegedly seeking to undermine social order, heteronormative values and promote “children's conversion.” By framing sexual and gender diversity as a societal threat, rather than a matter of human rights, these conspiracy beliefs may provide fertile ground for discrimination and opposition to legal protections aimed at safeguarding LGBTQ+ rights (Panerati et al. 2026).

Building on this literature, the present research examines the associations between perceived social isolation and the endorsement of conspiracy beliefs targeting LGBTQ+ people by focusing on conspiracy mentality as a general dispositional orientation that is conceptually distinct from, yet systematically related to, specific conspiracy beliefs (Bruder et al. 2013; Trella et al. 2024). Recent evidence further suggests that dispositional individual characteristics may be linked to conspiracy beliefs via conspiracy mentality, supporting its role as a psychological pathway through which broader vulnerabilities translate into specific conspiratorial narratives (Salvati 2025).

## 1 From Conspiracy Mentality to Specific Conspiracy Beliefs

*Conspiracy mentality* refers to the individuals' general disposition to believe in specific conspiracy beliefs. In this regard, Imhoff et al. (2022), propose that a conspiratorial mentality represents an enduring tendency to perceive events as the result of hidden malevolent schemes. Thus, conspiracy mentality reflects a broad conspiratorial mindset operating across contexts, whereas specific conspiracy beliefs concern concrete narratives targeting particular events or social groups (Sutton and Douglas 2020; Sutton et al. 2024). Empirical work further supports the conceptual and empirical separability of these constructs, while also highlighting their systematic association (Trella et al. 2024). *Conspiracy beliefs* can be seen as simple explanations of complex observable facts (e.g., the COVID‐19 pandemic), arising from the deliberate will of a small group of powerful and malevolent people to cover up information to suit their interests (Douglas and Sutton 2023). Accordingly, individuals with stronger conspiracy beliefs are more likely to interpret specific significant events as the result of hidden manoeuvers by powerful groups with harmful purposes. Therefore, although prior research has suggested that conspiracy mentality and specific conspiracy beliefs may also reinforce each other over time, theoretical models commonly conceptualize conspiracy mentality as a dispositional orientation that shapes the interpretative framework within which specific conspiratorial narratives are adopted (Bruder et al. 2013; Sutton and Douglas 2020).

Research has shown that conspiracy beliefs are often associated with prejudice and the need to maintain social hierarchies (Imhoff and Bruder 2014; Jolley et al. 2020). In this regard, the LGBTQ+ community has become a prominent target of conspiratorial narratives fuelled by public discourse that reinforces prejudice and the symbolic threat of LGBTQ+ people. From this perspective, conspiracy beliefs may serve as scapegoating narratives, attributing social change and perceived societal threats to stigmatized outgroups (van Prooijen 2021).

Although conspiracy beliefs against LGBTQ+ people show similarities with other conspiracy beliefs (e.g., a group of powerful people secretly plotting to gain an advantage), the LGBTQ+ conspiracy beliefs seem to represent a specific phenomenon with distinct features (Salvati, Pellegrini, De Cristofaro, Costacurta, and Giacomantonio 2024). According to these beliefs, LGBTQ+ individuals represent a “powerful lobby” whose aim is to undermine institutions, moral values and social order (Prearo 2023). This may be because LGBTQ+ individuals are perceived as nonconformists, and thus, they challenge traditional values and traditional family structures. From this perspective, LGBTQ+ individuals can be perceived as a destabilizing force in our society, and this perception, in turn, may lead individuals to adhere to conspiracy beliefs about the LGBTQ+ community, with the consequence of promoting further social exclusion and, sometimes, conflict. Among the consequences associated with conspiracy beliefs against the LGBTQ+ community, recent findings showed that they foster an increased detachment between the LGBTQ+ community and broader society (Gkinopoulos et al. 2024), more opposition toward the passing of protective laws (e.g., regarding the promotion of anti‐discrimination policies; De Cristofaro et al. 2025), and a more negative public opinion (Salvati, Pellegrini, De Cristofaro, Costacurta, and Giacomantonio 2024). These consequences may fuel and promote stereotypes, stigma, and prejudice against the LGBTQ+ community, hindering their rights and well‐being (Jolley et al. 2020).

## 2 The Relationship Between Perceived Social Isolation and Conspiracy Beliefs

From a psychological perspective, individuals may be particularly inclined to adopt a conspiratorial stance during periods characterized by uncertainty, instability, and perceived loss of control (Douglas et al. 2017). In such a context, conspiracy beliefs may provide an accessible framework for meaning‐making and the restoration of psychological coherence.

In this regard, recent evidence highlighted that individuals who feel more alienated from society might be more prone to adopt a conspiratorial thinking as a way to compensate for the lack of meaningfulness and the sense of belonging to society (Schnell et al. 2024). Consistent with these lines, individuals who experience persistent social isolation (characterized by objective vs. perceived lack of relationships, psychological barriers, physical barriers, and financial or environmental deficiencies; Nicholson 2009) often report a heightened level of mistrust toward official sources of information (van Prooijen 2022). Conspiratorial thinking may therefore serve multiple psychological functions, including the satisfaction of existential needs (e.g., feeling secure), epistemic needs (e.g., meaning‐making and reduction of uncertainty) and social needs (e.g., desire to protect a positive self‐image) (Douglas et al. 2017; Douglas and Sutton 2023; Salvati, Pellegrini, De Cristofaro, and Giacomantonio 2024). In this sense, a conspiratorial mindset may serve as a coping mechanism for individuals facing social exclusion (Imhoff 2024). Recent research further shows that loneliness (understood as alias perceived social isolation and unmet social needs; Courtin and Knapp 2017), as well as reduced social support are associated with a greater tendency to endorse conspiratorial thinking (Bertlich et al. 2025; Hettich et al. 2022; Cacioppo and Cacioppo 2018).

Furthermore, social isolation can lead someone to view others as untrustworthy or even potentially threatening, which can create a defensive and suspicious worldview (Qualter et al. 2010). This mindset may increase the appeal of conspiratorial narratives that identify outgroups as intentional sources of threats and social destabilization. This process can be further understood within the Appraisal Model of Conspiracy Theories (AMCT; Pummerer et al. 2024). This model suggests that when people feel particularly vulnerable (e.g., when they perceive themselves as socially isolated), they may be more inclined to see social change through threat‐based appraisals, eliciting negative emotions (e.g., anger and resentment). These feelings can facilitate the appeal of conspiratorial narratives, especially those that blame specific social groups (e.g., “the gay lobby”). Therefore, anti‐LGBTQ+ beliefs can be viewed as a specific example of a broader tendency to adopt a conspiratorial mindset driven by a personal appraisal. Thus, anti‐LGBTQ+ conspiracy beliefs might help individuals who feel socially excluded regain a sense of control (Douglas and Sutton 2023). Importantly, although Bierwiaczonek et al. (2024) focus on long‐term loneliness trajectory in relation to general conspiracy mentality rather than specific anti‐LGBTQ+ conspiracy beliefs, their findings highlight the role of persistent social disconnection in fostering a broader conspiratorial orientation, which may subsequently shape the endorsement of specific conspiracy narratives.

## 3 Current Study and Hypothesis

The present study investigated the relationships between perceived social isolation and anti‐LGBTQ+ conspiracy beliefs via the conspiracy mentality. Despite the growing body of research linking social isolation with general conspiracy mentality, relatively little is known about the specific processes that may account for the conspiracist beliefs against LGBTQ+ people. Building on prior theoretical and empirical work, the present study conceptualizes perceived social isolation as a contextual condition that may increase an individual's susceptibility to conspiratorial thinking by activating or strengthening a general conspiratorial mindset, rather than as a factor shaping stable personality characteristics. In this perspective, conspiracy mentality is understood as a relatively stable dispositional orientation whose expression may nevertheless vary as a function of social experiences, such as perceived social isolation.

In this framework, perceived social isolation is expected to be associated with higher levels of conspiracy mentality, which in turn facilitates the endorsement of specific conspiracy beliefs targeting LGBTQ+ people. Taken together, the associations lead to our main hypothesis: conspiracy mentality will partially mediate the relationship between perceived social isolation and endorsement of anti‐LGBTQ+ conspiracy beliefs, such that higher levels of perceived social isolation will be associated with higher endorsement of anti‐LGBTQ+ conspiracy beliefs both directly and indirectly through higher levels of conspiracy mentality.

## 4 Method

### 4.1 Participants and Procedures

Participants were recruited in Italy using a convenience sampling strategy. Specifically, data collection was conducted through an online survey disseminated through a snowball sampling procedure, starting from university students enrolled in the University course of Social Psychology by the corresponding author involved in data collection for university credits (i.e., they had to recruit at least 10 participants each). Data collection started in October 2024 and ended in December 2024. Participants were sent the link to the survey on the Qualtrics platform, and they were told that the research was about the investigation of the associations among some personal and interpersonal relational characteristics with some political and social opinions. Then, they were invited to read and sign informed consent. After completing the online questionnaire, participants were thanked and debriefed about the hypotheses of the study.

Inclusion criteria to participate in the research were: (a) being 18 years old; (b) being Italian; (c) not being an LGBTQ+ individual; (d) having correctly answered the attention check item requiring participants to select a specific response option (“other”) to demonstrate that they had read the instructions; (e) not having interrupted the questionnaire before the conclusion. A total of 1103 participants were recruited. All participants declared that they were 18 years old and Italian. However, 44 participants failed the attentional check item, 50 participants declared to be LGBTQ+, and 189 participants dropped out of the questionnaire before completing it. Thus, the final sample consisted of 820 Italian participants (*N*_men_ = 406, 49.5%; *N*_women_ = 414, 50.5%), ranging from 18 to 87 years old, *M* = 42.5, SD = 16.5. See Table 1 for the sample's descriptives.

**TABLE 1:** Sample's characteristics.

Variable	*N*	%
Sex and gender		
Male/Man	406	49.5
Female/Woman	414	50.5
Sexual orientation		
Exclusively heterosexual	751	91.6
Predominantly heterosexual	69	8.4
Residency		
Northern Italy	690	84.1
Central Italy	18	2.2
Southern Italy and Islands	101	12.3
Abroad	11	1.3
Education level		
Primary School Diploma	13	1.6
Middle School Diploma	152	18.5
High School Diploma	462	56.3
Bachelor's Degree	79	9.6
Master's Degree	93	11.3
PhD or Higher Specialization	21	2.6

The study was not preregistered. To ensure transparency, all inclusion and exclusion criteria, hypothesis and analytic decisions are reported. The anonymized dataset and study materials are publicly available at the following link: https://osf.io/am7nq/overview?view_only=c192c239c7744bf787929208419c24e1.

#### 4.1.1 A Priori Power Analysis

A priori power analysis was conducted to determine the minimum sample size required for the study. To estimate an adequate sample size to detect the indirect effect, a Monte Carlo a priori power analysis for indirect effects was run by the free online software ‘ShinyApp’ (Schoemann et al. 2017). We have set the following option: Model: one mediator; Target Power: 0.80; Minimum *N* = 50; Maximum *N* = 800; Sample Size Steps: 25; # of Replications: 5000; Monte Carlo Draws per Rep: 20000; Random Seed: 1234; Confidence Level (%): 95; Input Method: Correlations. The expected correlation between conspiracy mentality (*M*) and anti‐LGBTQ+ conspiracy beliefs (*Y*) was calculated through the average correlation among the three ones found in previous studies [Study 1 by Salvati, Pellegrini, De Cristofaro, and Giacomantonio (2024)—*r*_1_ = 0.21; Study 1 and Study 2 by Salvati, Pellegrini, De Cristofaro, Costacurta, and Giacomantonio (2024)—*r*_2_ = 0.28; *r*_3_ = 0.33]. Thus *r*_MY_ = 0.30 was set. Instead, lacking previous literature which investigated the specific associations of the social connection scale (*X*) with conspiracy mentality and anti‐LGBTQ+ conspiracy beliefs, respectively, a parsimonious small correlation was set for both: *r* = 0.10. The results of the power analysis indicated a minimum sample of 725 participants to detect the indirect effect.

### 4.2 Measures

#### 4.2.1 Sociodemographic Section

One single item asked participants' biological sex, providing three possible options (Male, Female, Other), whereas another single item asked participants' gender, through five possible options (Man, Woman, Trans*, Agender, Other). Then, participants were invited to indicate their age, nationality, residency (Northern Italy, Central Italy, Southern Italy and Islands, Abroad), and educational level. Subsequently, participants reported their sexual orientation through a single item with six options (i.e., *exclusively heterosexual, mainly heterosexual, bisexual, mainly homosexual, exclusively homosexual, other*).

#### 4.2.2 Political Orientation

We used a 7‐point single‐item Likert scale that asked participants to select their political orientation from 1 = *extremely left* to 7 = *extremely right*.

#### 4.2.3 Religiosity

Participants were administered a scale of five items on a 5‐point Likert scale, from 1 = *not at all* to 5 = *completely*, to measure their level of religiosity (Cronbach's alpha = 0.89). An example item was: “Currently, I consider myself a religious person”. The total score was computed by averaging the scores of all the items so that higher levels corresponded to higher religiosity.

#### 4.2.4 Perceived Social Isolation

In order to have a measure of perceived social isolation, we used the UBC State Social Connection Scale (Lok and Dunn 2023). Participants were invited to report their levels of agreement, from 1 = *strongly disagree* to 7 = *strongly agree*, to 10 items which asked them to think about how they felt during last week (Cronbach's alpha = 0.88). Example items were: “I didn't feel related to most people” or “I felt a strong bond with other people (reversed)”. After having appropriately reversed some items, the total score was calculated through the average of the 10 items, so that higher scores on the scale corresponded to higher levels of social isolation.

#### 4.2.5 Conspiratorial Mentality

We used the Conspiracy Mentality Questionnaire (Bruder et al. 2013), which asked participants to respond to five items, indicating the degree of probability for each of them on an 11‐point Likert scale, from 0% = *certainly not*, to 100% = *certainly yes* (Cronbach's alpha = 0.87). An example item was: “I think that government agencies closely monitor all citizens”. The total score was calculated by averaging the 5 items, so that high levels of the scale corresponded to high levels of conspiracy mentality.

#### 4.2.6 Anti‐LGBTQ+ Conspiracy Beliefs

Participants responded to the gender ideology and LGBTQ+ lobby conspiracies beliefs (GILC) scale (Salvati, Pellegrini, De Cristofaro, and Giacomantonio 2024). Such a tool has nine items on a 5‐point Likert scale, ranging from 1 = *totally disagree* to 5 = *totally agree* (Cronbach's alpha = 0.95). An example of two items of the scale are the following: “There are very powerful LGBT people who manage to influence the decisions of the Parliament and the Government, to the detriment of other citizens*”; “*Some very powerful people want to spread ‘gender ideology’ in schools to indoctrinate children”. The final score was computed through averaging the nine items, so that high scores in the GILC scale corresponded to higher levels of anti‐LGBTQ+ conspiracy beliefs.

#### 4.2.7 Sexual Prejudice

We used a brief, subtle, and modern measure of sexual prejudice, named denial of discrimination against LGBTQ+ people (Salvati, Pellegrini, De Cristofaro, and Giacomantonio 2024). Such a tool assessed participants' beliefs that discrimination against LGBTQ+ people is no longer an issue in our society. Participants responded to 3 items (e.g., d*iscrimination against LGBTQ+ people is no longer a problem in this country*) on a 5‐point Likert scale, ranging from 1 = *totally disagree* to 5 = *totally agree* (Cronbach's alpha = 0.78). The final score was computed by the mean of the three items, so that higher scores corresponded to higher levels of sexual prejudice against LGBTQ+ people.

### 4.3 Strategy Analysis

**FIGURE 1:**
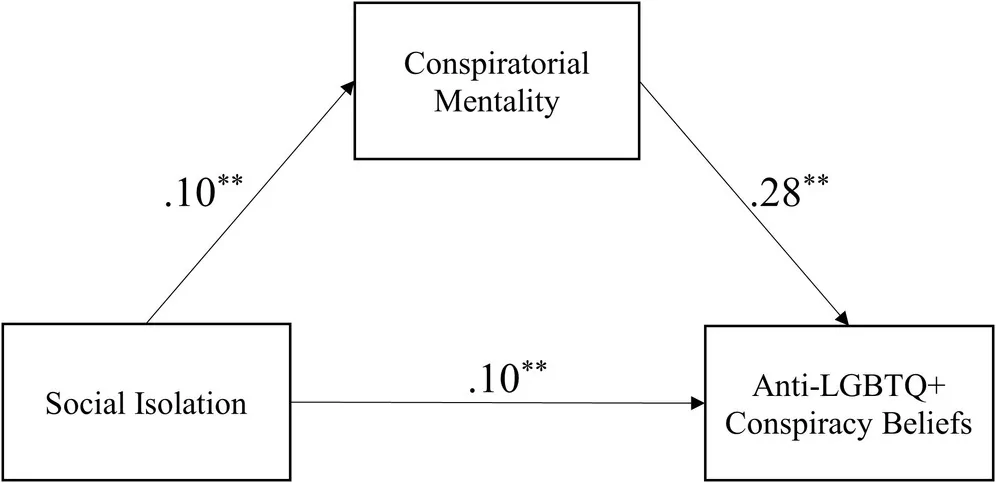
Mediation model testing the indirect association of social isolation with anti‐LGBTQ+ conspiracy beliefs via conspiratorial mentality (*N* = 820). Standardized coefficients are reported. The model includes gender, age, religiosity, political orientation, and sexual prejudice as covariates.

We have run preliminary analyses to test that multicollinearity and normality were not an issue. Thus, we calculated skewness and kurtosis, based on the cutoff of < |3|, and we have run correlations considering the maximum value of |0.80| for the absence of multicollinearity. The main analyses consisted of a regression mediation model (Figure 1) to test our research hypotheses. Specifically, the social connection scale entered the predictor (*X*), the conspiracy mentality entered as mediator (*M*), and the anti‐LGBTQ+ conspiracy beliefs were the dependent variable (*Y*). Such a mediation model was run both including and not including gender, age, educational level, religiosity, political orientation, and denial of discrimination as covariates. All the analyses were run using SPSS, version 29, and its macro PROCESS. The significance of the indirect association was computed through the percentile bootstrap method with 5000 samples, whereas we have set the confidence intervals at 95%.

## 5 Results

The results of preliminary analyses (Table 2). The results of preliminary analyses (Table 2) showed that skewness and kurtosis values were generally within acceptable ranges, with only one value slightly exceeding |1| (George and Mallery 2024). Furthermore, none of the correlations among the study variables exceeded commonly accepted cutoff |0.80|, suggesting no issues of multicollinearity (Tabachnick and Fidell 2019). Descriptive statistics for all the study variables, including means and standard deviations, are reported in Table 2. In this regard, correlation results provided preliminary support to our hypotheses, showing that higher levels of social isolation were associated both with higher levels of conspiracy mentality, *r* = 0.09, *p* < 0.01, and with higher levels of anti‐LGBTQ+ conspiracy beliefs, *r* = 0.15, *p* < 0.01, although these associations were small in magnitude. In contrast, conspiracy mentality showed a stronger positive association with anti‐LGBTQ+ conspiracy beliefs, *r* = 0.31, *p* < 0.01, with a medium effect size. Overall, this pattern of bivariate correlations is consistent with the hypothesized relationship and provides the basis for the subsequent mediation analyses.

**TABLE 2:** Descriptives and correlations (*N* = 820).

	Gender	Age	Education level	Political orientation	Religiosity	Social isolation	Conspiratorial mentality	Anti‐LGBTQ + conspiracy beliefs	Sexual prejudice
Gender	1								
Age	−0.07*	1							
Education level	0.02	−0.17**	1						
Political orientation	−0.10**	0.16**	−0.18**	1					
Religiosity	0.09**	0.28**	−0.06	0.17**	1				
Social isolation	−0.06	−0.11**	−0.04	0.05	−0.04	1			
Conspiratorial mentality	0.15**	0.09*	−0.21**	0.12**	0.03	0.09**	1		
Anti‐LGBTQ+ Conspiracy beliefs	−0.06	0.16**	−0.18**	0.35**	0.27**	0.15**	0.31**	1	
Sexual prejudice	−0.15**	0.15**	−0.08*	0.28**	0.20**	0.04	−0.02	0.38**	1
*M*	—	42.47	—	4.05	2.58	4.89	7.34	1.98	2.19
SD	—	16.53	—	1.40	1.12	1.18	2.14	1.03	0.94
Skewness	—	0.05	—	−0.01	0.58	−0.41	−0.40	1.02	0.72
Kurtosis	—	−1.10	—	−1.00	−0.69	−0.27	−0.39	0.28	0.15

*Note:* *p < 0.05; **p < 0.01; Gender: 1 = Men; 2 = Women; Political orientation: 1 = extremely left; 7 = extremely right.

The results of the regression mediation model (Table 3) were in line with our hypotheses, both including and not including gender, age, educational level, religiosity, political orientation, and denial of discrimination as covariates. Here, we report all the results of the model, including all the covariates, believing that they would strengthen the results. The model explained a significant proportion of anti‐LGBTQ+ conspiracy beliefs variance, *R* ^*2*^ = 32.97%, *F*(8,811) = 49.87, *p* < 0.001.

**TABLE 3:** Regression model (*N* = 820).

Effect			*b*	*β*	SE	95% confidence interval		*t*	*p*
						Lower	Upper		
Social isolation	(a) →	Conspiratorial mentality	0.19	0.10	0.06	0.065	0.306	3.02	0.003
Conspiratorial mentality	(b) →	Anti‐LGBTQ+ conspiracy beliefs	0.13	0.28	0.01	0.105	0.162	9.20	< 0.001
Social isolation	(c) →	Anti‐LGBTQ+ conspiracy beliefs	0.09	0.10	0.03	0.039	0.140	3.51	< 0.001
Total effect	c + a × b		0.11	0.13	0.03	0.062	0.167	4.29	< 0.001
Indirect effect	a × b		0.02	0.03	0.01	0.009	0.042		

*Note:* The results include gender, age, education, religiosity, political orientation, and sexual prejudice as covariates.

Specifically, the results showed that the positive association between social isolation and anti‐LGBTQ+ conspiracy beliefs resulted significant, both before, *β* = −0.13, *p* < 0.001, and after adding conspiracy mentality as a mediator to the model, *β* = −0.10, *p* < 0.001. Furthermore, higher social isolation was related to higher conspiracy mentality, *β* = −0.10, *p* = 0.003. Also, higher levels of conspiracy mentality were related to higher levels of anti‐LGBTQ+ conspiracy beliefs, *β* = 0.28, *p* < 0.001. Finally, in line with our hypothesis, the results showed that the mediating association of social isolation with anti‐LGBTQ+ conspiracy beliefs via conspiracy mentality resulted significant, *β* = −0.03, 95% CI [−0.048, −0.010].

## 6 Discussion

Our study aims to advance our understanding of the psychosocial processes associated with the endorsement of anti‐LGBTQ+ conspiracy beliefs by identifying relational patterns among perceived social isolation, conspiracy mentality, and anti‐LGBTQ+ conspiracy beliefs. Particularly, the findings of our study highlight the complex relationship between perceived social isolation, conspiracy mentality, and the endorsement of anti‐LGBTQ+ conspiracy beliefs. While previous research has mainly emphasized individual psychological traits in explaining conspiratorial mentality, the present work addresses perceived social isolation as a relational and context‐sensitive psychological experience, rather than a stable individual characteristic. Rather than introducing a novel construct, the present study contributes by extending existing research to examine perceived social isolation as a psychosocial condition that reflects an individual's subjective appraisal of their social embeddedness and connectedness. Indeed, while previous studies have mainly focused on causes and the consequences of general conspiracy beliefs (Uscinski et al. 2022), much less attention has been given to how these dynamics work when conspiracies specifically target marginalized groups, such as the LGBTQ+ community.

For this reason, the present study aimed to shed light on the phenomenon by showing a positive relationship between perceived social isolation and the endorsement of anti‐LGBTQ+ conspiracy beliefs. Findings showed that participants reporting a higher level of perceived social isolation were more likely to report a greater endorsement of anti‐LGBTQ+ conspiracy beliefs. This relationship is in line with previous research suggesting that socially isolated individuals are more likely to experience existential anxiety, and thus to adopt simplified explanations for complex social phenomena (Miceli and Castelfranchi 2005). These simplified explanations may be functional for individuals experiencing social exclusion, helping them find meaning and a sense of coherence in situations they perceive as threatening or disordered (van Prooijen 2017).

Furthermore, our findings showed that a higher level of perceived social isolation was positively associated with a conspiracy mentality. Indeed, the results are consistent with the idea that experiences of isolation and social disconnection may nourish a generalized attitude of suspicion and distrust (Cacioppo and Cacioppo 2018; Schnell et al. 2024). The experience of social isolation tends to make people more vulnerable to alternative, nonofficial explanations of phenomena, even when such interpretations appear to be conspiratorial or implausible (Imhoff 2024). In this sense, a conspiratorial mindset may represent a coping mechanism for individuals facing social exclusion (Marchlewska et al. 2021).

It is interesting to notice that higher levels of conspiratorial mentality were related to higher levels of anti‐LGBTQ+ conspiracy beliefs. This finding is consistent with previous studies suggesting that a conspiratorial mindset often manifests through specific narratives, particularly those aimed at preserving traditional social norms and hierarchies (Imhoff and Bruder 2014; Jolley et al. 2020). Narratives about LGBTQ+ individuals may include their representation as part of an influential group perceived as challenging conventional social norms and destabilizing traditional values (Salvati, Pellegrini, De Cristofaro, Costacurta, and Giacomantonio 2024).

Finally, we found an indirect association between social isolation and anti‐LGBTQ+ conspiracy beliefs, via the conspiracy mentality. Indeed, we found evidence concerning this relationship, confirming that higher levels of perceived social isolation were associated with higher levels of conspiracy mentality, which, in turn, were related to a higher endorsement of conspiracy beliefs against LGBTQ+ people. It is worth noting that these findings remained consistent even after controlling for potentially confounding variables (gender, age, religiosity, education level, political orientation, and denial of discrimination), confirming that the relationship is robust across demographic variables and that the observed effects are not driven by those variables. At the same time, given the cross‐sectional nature of the present study, these indirect associations should be interpreted with caution. Cross‐sectional studies do not allow for causal inference and cannot establish temporal precedence among variables. Although the present model is theoretically grounded, alternative explanations remain plausible, including reciprocal or reinforcing processes whereby conspiracy mentality or conspiracy beliefs themselves may contribute to increased perceptions of social isolation over time. Longitudinal and experimental design will therefore be necessary to disentangle the directionality of these associations and to test whether perceived social isolation precedes changes in conspiracy mentality, or whether these processes mutually influence one another.

For individuals experiencing social isolation, conspiracy beliefs may represent both a way of giving meaning to an unpredictable world and a way to belong to something, through the affiliation with other individuals who share similar perspectives and views (Bertlich et al. 2025; Biddlestone et al. 2025; van Prooijen 2022). Indeed, as highlighted by van Prooijen (2022), conspiracy beliefs may fulfill certain psychological needs, providing individuals with transient psychological comfort, such as helping them to make sense of unpredictable and/or complex events by offering a simplified explanation, although the effects on well‐being are negative in the long term. This kind of immediate psychological reward may help explain why conspiracy beliefs persist: they would not only be due to cognitive biases or misinformation, but they would also represent coping mechanisms, particularly for those individuals who feel disconnected from others, and would offer a form of psychological comfort. These individuals, by adopting such beliefs, may feel a sense of group identity, offering meaning and a sense of belonging in an otherwise uncertain social environment. This is why the social and clinical implications of our study are considerable, considering that conspiracy beliefs can have detrimental consequences, including reinforcement of harmful stereotypes, stigmatization and reduced support for anti‐discrimination laws (De Cristofaro et al. 2025; Gkinopoulos et al. 2024). In this regard, it is important to expand the understanding concerning the endorsement of anti‐LGBTQ+ conspiracy beliefs to social processes (Panerati and Salvati 2025), going beyond individual characteristics. In this regard, the individual's social context may represent a useful approach to highlight the factors that produce fertile ground for the endorsement of those beliefs.

### 6.1 Limitations, Future Directions, and Social Impact

This study also presents some limitations that must be acknowledged. Firstly, the cross‐sectional design does not support causal relationships. Future directions might include longitudinal studies to determine whether social isolation precedes the development of conspiracy mentality or whether these variables interact in a different way over time, for example, if such beliefs themselves contribute to social isolation. Additionally, future studies could investigate whether similar mediating mechanisms apply to conspiracies targeting other marginalized groups, besides the LGBTQ+ population.

Secondly, the data were drawn from a convenience sample, which limits the generalizability of the findings: the sample may not adequately represent the broader population in terms of key variables such as age, political orientation, and education. Future studies could employ more representative sampling strategies to assess the consistency of these findings better.

Furthermore, the present study was conducted in an Italian context using a convenience sample, which limits the generalizability of the findings. Future studies should examine whether the observed associations replicate in different national contexts characterized, for instance, by different levels of institutional support for LGBTQ+ rights. In addition, the current study focused on the general population. Future studies could extend this work by taking into account how conspiracy beliefs targeting LGBTQ+ people might be internalized from LGBTQ+ people themselves, for instance, concerning phenomena such as internalized homophobia.

Thirdly, the study relied exclusively on self‐report measures, which may limit its conclusions: although self‐report instruments are widely used in psychological research, they may have some limitations. Responses may be influenced by variables such as social desirability bias, and this is particularly relevant when exploring sensitive topics, such as attitudes toward marginalized groups, such as the LGBTQ+ population. In addition, self‐reported data may detect more subjective perspectives rather than observable behavior in a real‐world context, which may result in incoherence. Future research could employ additional data sources in order to validate the conclusions drawn from self‐report instruments. Related to this, a further limitation concerns the operationalization of perceived social isolation. Indeed, in the present study, social isolation was assessed in terms of perceived social isolation, reflecting an individual's subjective evaluations of their social connectedness. Although this approach is theoretically meaningful and widely used, it does not allow for capturing more objective indicators concerning social isolation, such as the size of one's social network, frequency of social interaction, time spent with significant others, and so on. Future research could benefit from integrating both subjective and objective measures of social isolation to examine whether perceived and structural aspects of social disconnectedness differentially relate to conspiracy mentality and conspiracy beliefs.

Finally, although gender did not show a significant association with anti‐LGBTQ+ beliefs in the present study, future research may nevertheless benefit from further exploring the role of gender‐related processes in this domain. In particular, it may be valuable to investigate whether gender identity, gender norm adherence, or gender‐related existential concerns (such as threatened masculinity) might interact with social isolation and conspiracy mentality in shaping susceptibility to anti‐LGBTQ+ conspiracy beliefs. Such an approach could help shed light on whether gender operates as a conditional factor (rather than a predictor) influencing how social isolation influences conspiracy mentality and translates into conspiratorial thinking targeting gender and sexual minorities.

Upcoming efforts may empirically test whether and how initiatives aimed at reducing social isolation and loneliness are associated with changes in conspiracy‐related mentality and beliefs, for instance, through longitudinal or experimental designs that allow for causal inference.

These informational initiatives would not only tackle the psychological basis of conspiracy thinking but would also promote understanding of others and empathy, thereby alleviating the social damage these beliefs may cause and enhancing individual well‐being. Educating the public in schools, at work and in other environments on these variables may promote a deeper understanding of how social isolation can affect beliefs, perceptions, and emotions and its detrimental effects. At this stage, however, any implications for applied interventions or public policy remain speculative given the correlational nature of the present findings. Nevertheless, existing theoretical and empirical work (Imhoff 2024) suggests that social connectedness and perceived belonging represent relevant psychosocial dimensions in understanding vulnerability to conspiracy thinking. Future research may therefore benefit from examining how broader social environments, relational experiences, and perception of connectedness relate to conspiracy mentality and specific conspiracy beliefs. In this sense, innovative approaches to fostering social connections and reducing the experience of social isolation may represent promising avenues for future investigation and developing strategies to tackle the phenomenon.

## 7 Conclusion

The current study offers a novel perspective on the interplay among conspiracy mentality, endorsement of conspiracy beliefs against LGBTQ+ people and perceived social isolation. By examining these, we found that feeling socially isolated is associated with greater endorsement of anti‐LGBTQ+ conspiracy beliefs both directly and indirectly through higher levels of conspiracy mentality. While we cannot draw causal conclusions from these findings, they do help deepen our understanding of the psychological factors that might lead to the rise of anti‐LGBTQ+ conspiracy beliefs. Therefore, addressing feelings of social isolation may represent an important aspect to consider in future research and prevention strategies, particularly when aiming to understand vulnerability to conspiratorial narratives targeting sexual and gender minorities.

## Author Contributions

**S.P.:** writing – original draft, visualization. **B.B.:** conceptualization, methodology, writing – original draft, writing – review and editing. **M.Y.E.:** writing – review and editing. **M.S.:** conceptualization, data curation, formal analysis, funding acquisition, investigation, methodology, project administration, supervision.

## Funding

This study was funded by “PRIN 2022” by the Italian Ministry of University and Research (MUR), won by the corresponding author (PI of the CUP Project: B53D23019360001; Prot no. 1060 of 17/07/2023; PNRR for the Mission 4, investment 1.1., funded by the European Union – NextGenerationEU).

## Ethics Statement

The research has been approved by the Bioethics Committee at the University of Verona, Italy (ID: Prot. no. 15395 del 08/01/2025). The research complied with the Declaration of Helsinki and the AIP ethical code (2023).

## Consent

Participants read the informed consent and provided a written agreement before taking part in the studies. In compliance with established ethical principles, participation was voluntary and completely anonymous.

## Conflicts of Interest

The authors declare no conflicts of interest.

## Data Availability

Herewith, we state that the data were collected in a manner consistent with ethical standards for treating human subjects. The anonymized dataset and study materials are publicly available at the following link: https://osf.io/am7nq/overview?view_only=c192c239c7744bf787929208419c24e1.

## References

[sjop70111-bib-0001] Bayar, M. C. 2025. “Partisan and Non‐Partisan Conspiracy Theories' Diverging Effects on Political Participation.” Electoral Studies 95: 102920. 10.1016/j.electstud.2025.102920.

[sjop70111-bib-0002] Bertlich, T. , A. K. Bräscher , S. Germer , M. Witthöft , and R. Imhoff . 2025. “Owners of a Conspiratorial Heart? Investigating the Longitudinal Relationship Between Loneliness and Conspiracy Beliefs.” British Journal of Social Psychology 64, no. 2: e12865. 10.1111/bjso.12865. 39976276 PMC11840883

[sjop70111-bib-0003] Biddlestone, M. , R. Green , K. M. Douglas , F. Azevedo , R. M. Sutton , and A. Cichocka . 2025. “Reasons to Believe: A Systematic Review and Meta‐Analytic Synthesis of the Motives Associated With Conspiracy Beliefs.” Psychological Bulletin 151, no. 1: 48–87. 10.1037/bul0000463. 39913483

[sjop70111-bib-0004] Bierwiaczonek, K. , S. Fluit , T. von Soest , M. Hornsey , and J. Kunst . 2024. “Loneliness Trajectories Over Three Decades Are Associated With Conspiracist Worldviews in Midlife.” Nature Communications 14: 3629. 10.1038/s41467-024-47113-x. PMC11059163 38684667

[sjop70111-bib-0005] Bruder, M. , P. Haffke , N. Neave , N. Nouripanah , and R. Imhoff . 2013. “Measuring Individual Differences in Generic Beliefs in Conspiracy Theories Across Cultures: Conspiracy Mentality Questionnaire.” Frontiers in Psychology 4: 225. 10.3389/fpsyg.2013.00225. 23641227 PMC3639408

[sjop70111-bib-0006] Cacioppo, J. T. , and S. Cacioppo . 2018. “Loneliness in the Modern Age: An Evolutionary Theory of Loneliness (ETL).” Advances in Experimental Social Psychology 58: 127–197. 10.1016/bs.aesp.2018.03.003.

[sjop70111-bib-0007] Courtin, E. , and M. Knapp . 2017. “Social Isolation, Loneliness and Health in Old Age: A Scoping Review.” Health & Social Care in the Community 25, no. 3: 799–812. 10.1111/hsc.12311. 26712585

[sjop70111-bib-0008] De Cristofaro, V. , M. Costacurta , V. Pellegrini , M. Giacomantonio , and M. Salvati . 2025. “LGBTQ+ Conspiracy Beliefs and Collective Actions: Factors and Processes That (de)motivate Support for LGBTQ+ Equality.” Analyses of Social Issues and Public Policy 25: e70001. 10.1111/asap.70001.

[sjop70111-bib-0009] Douglas, K. M. , and R. M. Sutton . 2023. “What Are Conspiracy Theories? A Definitional Approach to Their Correlates, Consequences, and Communication.” Annual Review of Psychology 74, no. 1: 271–298. 10.1146/annurev-psych-032420-031329. 36170672

[sjop70111-bib-0010] Douglas, K. M. , R. M. Sutton , and A. Cichocka . 2017. “The Psychology of Conspiracy Theories.” Current Directions in Psychological Science 26, no. 6: 538–542. 10.1177/0963721417718261. 29276345 PMC5724570

[sjop70111-bib-0011] European Commission . 2020. “Union of Equality: LGBTIQ Equality Strategy 2020–2025.” https://commission.europa.eu/system/files/2020‐11/lgbtiq_strategy_2020‐2025_en.pdf.

[sjop70111-bib-0012] George, D. , and P. Mallery . 2024. “IBM SPSS Statistics 29 Step by Step: A Simple Guide and Reference.” Routledge, 1–440. 10.4324/9781032622156.

[sjop70111-bib-0013] Gkinopoulos, T. , M. Teresi , C. Ballone , H. Çakmak , M. G. Pacilli , and S. Pagliaro . 2024. “Religiosity and Social Distance From LGBTQI+ People: The Mediating Role of Gender and LGBTQI+ Conspiracy Beliefs.” Sexuality Research & Social Policy 21: 912–920. 10.1007/s13178-024-00962-z.

[sjop70111-bib-0014] Graeupner, D. , and A. Coman . 2017. “The Dark Side of Meaning‐Making: How Social Exclusion Leads to Superstitious Thinking.” Journal of Experimental Social Psychology 69: 218–222. 10.1016/j.jesp.2016.10.003.

[sjop70111-bib-0015] Hettich, N. , M. E. Beutel , M. Ernst , et al. 2022. “Conspiracy Endorsement and Its Associations With Personality Functioning, Anxiety, Loneliness, and Sociodemographic Characteristics During the COVID‐19 Pandemic in a Representative Sample of the German Population.” PLoS One 17, no. 1: e0263301. 10.1371/journal.pone.0263301. 35089987 PMC8797225

[sjop70111-bib-0016] Hornsey, M. J. , K. Bierwiaczonek , K. Sassenberg , and K. M. Douglas . 2023. “Individual, Intergroup and Nation‐Level Influences on Belief in Conspiracy Theories.” Nature Reviews Psychology 2, no. 2: 85–97. 10.1038/s44159-022-00133-0. PMC9685076 36467717

[sjop70111-bib-0017] ILGA‐Europe . 2024. “Annual Review of the Human Rights Situation of LGBTI People in Europe and Central Asia.” ILGA‐Europe. https://www.ilga‐europe.org/report/annual‐review‐2024/.

[sjop70111-bib-0018] Imhoff, R. 2024. “Connecting Conspiracy Beliefs and Experiences of Social Exclusion.” In Exclusion and Extremism: A Psychological Perspective, edited by M. Pfundmair , A. H. Hales , and K. D. Williams , 287–307. Cambridge University Press.

[sjop70111-bib-0019] Imhoff, R. , T. Bertlich , and M. Frenken . 2022. “Tearing Apart the ‘Evil’ Twins: A General Conspiracy Mentality Is Not the Same as Specific Conspiracy Beliefs.” Current Opinion in Psychology 46: 101349. 10.1016/j.copsyc.2022.101349. 35537265

[sjop70111-bib-0020] Imhoff, R. , and M. Bruder . 2014. “Speaking (Un‐) Truth to Power: Conspiracy Mentality as a Generalised Political Attitude.” European Journal of Personality 28, no. 1: 25–43. 10.1002/per.1930.

[sjop70111-bib-0021] Jolley, D. , R. Meleady , and K. M. Douglas . 2020. “Exposure to Intergroup Conspiracy Theories Promotes Prejudice Which Spreads Across Groups.” British Journal of Psychology 111, no. 1: 17–35. 10.1111/bjop.12385. 30868563 PMC7004178

[sjop70111-bib-0022] Jolley, D. , C. R. Seger , and R. Meleady . 2023. “More Than a Prejudice Reduction Effect: Positive Intergroup Contact Reduces Conspiracy Theory Beliefs.” European Journal of Social Psychology 53, no. 6: 1262–1275. 10.1002/ejsp.2973.

[sjop70111-bib-0023] Lok, I. , and E. Dunn . 2023. “The UBC State Social Connection Scale: Factor Structure, Reliability, and Validity.” Social Psychological and Personality Science 14, no. 7: 835–844. 10.1177/19485506221132090. 37545485 PMC10396795

[sjop70111-bib-0024] Marchlewska, M. , R. Green , A. Cichocka , Z. Molenda , and K. M. Douglas . 2021. “From Bad to Worse: Avoidance Coping With Stress Increases Conspiracy Beliefs.” British Journal of Social Psychology 61, no. 2: 532–549. 10.1111/bjso.12494. 34462919

[sjop70111-bib-0025] Miceli, M. , and C. Castelfranchi . 2005. “Anxiety as an “Epistemic” Emotion: An Uncertainty Theory of Anxiety.” Anxiety, Stress, and Coping: An International Journal 18, no. 4: 291–319. 10.1080/10615800500209324.

[sjop70111-bib-0026] Mouafo, A. V. D. 2023. “The Denial of Homosexual Identity as a Mediator of the Link Between Beliefs in a Gay Conspiracy and Hostile Intentions Towards LGBTQ People in a Highly Heteronormative Context: The Case of Cameroon.” International Journal of Psychology and Behavioral Sciences 13, no. 2: 29–37. 10.5923/j.ijpbs.20231302.01.

[sjop70111-bib-0027] Nicholson, N. R. 2009. “Social Isolation in Older Adults: An Evolutionary Concept Analysis.” Journal of Advanced Nursing 65, no. 6: 1342–1352. 10.1111/j.1365-2648.2008.04959.x. 19291185

[sjop70111-bib-0028] Panerati, S. , V. De Cristofaro , V. Pellegrini , and M. Salvati . 2026. “Conspiratorial Threat and Intergroup Boundaries: How Exposure to Anti‐LGBTQ+ Narratives Shapes Beliefs, Identity, and Civic Engagement.” Group Process and Intergroup Relations.

[sjop70111-bib-0029] Panerati, S. , and M. Salvati . 2025. “The More Positive Intergroup Contacts You Have, the Less LGBTQ+ Conspiracies Beliefs You Will Report: The Role of Knowledge, Anxiety, and Empathy.” British Journal of Social Psychology 64, no. 2: e12866. 10.1111/bjso.12866. 39953817 PMC11829741

[sjop70111-bib-0030] Phadke, S. , M. Samory , and T. Mitra . 2021. “What Makes People Join Conspiracy Communities? Role of Social Factors in Conspiracy Engagement.” Proceedings of the ACM on Human‐Computer Interaction 4: 1–30. 10.1145/3432922.

[sjop70111-bib-0031] Poon, K. T. , Z. Chen , and W. Y. Wong . 2020. “Beliefs in Conspiracy Theories Following Ostracism.” Personality and Social Psychology Bulletin 46, no. 8: 1234–1246. 10.1177/0146167219898944. 31928312

[sjop70111-bib-0032] Prearo, M. 2023. “The Anti‐Gender and Gender‐Critical Roots of the Italian Anti‐Trans Parent Activism.” Journal of Diversity and Gender Studies 10, no. 2: 115–117. 10.21825/digest.89996.

[sjop70111-bib-0034] Pummerer, L. , T. Gkinopoulos , K. M. Douglas , D. Jolley , and K. Sassenberg . 2024. “The Appraisal Model of Conspiracy Theories (AMCT): Highlighting Core Concepts and Potential Extensions.” Psychological Inquiry 35, no. 3–4: 233–242. 10.1080/1047840X.2025.2454118. 40129957 PMC11931513

[sjop70111-bib-0035] Qualter, P. , S. L. Brown , P. Munn , and K. J. Rotenberg . 2010. “Childhood Loneliness as a Predictor of Adolescent Depressive Symptoms: An 8‐Year Longitudinal Study.” European Child & Adolescent Psychiatry 19, no. 6: 493–501. 10.1007/s00787-009-0059-y. 19777287

[sjop70111-bib-0036] Salvati, M. 2025. “Dispositional Mindfulness, Conspiracy Mentality, and Generic Conspiracy Beliefs: Preliminary Empirical Evidence of a Mediational Model.” Personality and Individual Differences 238: 113090. 10.1016/j.paid.2025.113090.

[sjop70111-bib-0037] Salvati, M. , M. Giacomantonio , V. Pellegrini , V. De Cristofaro , and L. Leone . 2022. “Conspiracy Beliefs of Italian Voters for Populist Parties: The Moderated Mediational Role of Political Interest and Ideological Attitudes.” Acta Psychologica 223: 103508. 10.1016/j.actpsy.2022.103508. 35065530

[sjop70111-bib-0038] Salvati, M. , V. Pellegrini , V. De Cristofaro , M. Costacurta , and M. Giacomantonio . 2024. “Antecedent Ideological Profiles and Negative Socio‐Political Outcomes of LGBTQ+ Conspiracy Beliefs.” Sexuality Research & Social Policy 21, no. 3: 899–911. 10.1007/s13178-024-00949-w.

[sjop70111-bib-0039] Salvati, M. , V. Pellegrini , V. De Cristofaro , and M. Giacomantonio . 2024. “What Is Hiding Behind the Rainbow Plot? The Gender Ideology and LGBTQ+ Lobby Conspiracies (GILC) Scale.” British Journal of Social Psychology 63, no. 1: 295–318. 10.1111/bjso.12678. 37606152

[sjop70111-bib-0040] Schnell, T. , R. Viviani , C. Lenz , and H. Krampe . 2024. “When Alienated From Society, Conspiracy Theory Belief Gives Meaning to Life.” Heliyon 10, no. 14: e34557. 10.1016/j.heliyon.2024.e34557. 39149052 PMC11324984

[sjop70111-bib-0041] Schoemann, A. M. , A. J. Boulton , and S. D. Short . 2017. “Determining Power and Sample Size for Simple and Complex Mediation Models.” Social Psychological and Personality Science 8, no. 4: 379–386. 10.1177/1948550617715068.

[sjop70111-bib-0042] Sutton, R. M. , and K. M. Douglas . 2020. “Conspiracy Theories and the Conspiracy Mindset: Implications for Political Ideology.” Current Opinion in Behavioral Sciences 34: 118–122. 10.1016/j.cobeha.2020.02.015.

[sjop70111-bib-0043] Sutton, R. M. , K. M. Douglas , and C. Trella . 2024. “Conspiracy Mentality Versus Belief in Conspiracy Theories.” Zeitschrift für Psychologie 232, no. 1: 50–54. 10.1027/2151-2604/a000549.

[sjop70111-bib-0044] Tabachnick, B. G. , and L. S. Fidell . 2019. Using Multivariate Statistics. 7th ed. Pearson.

[sjop70111-bib-0045] Trella, C. , R. M. Sutton , and K. M. Douglas . 2024. “Semantic and Causal Relations Between the Conspiracy Mentality and Belief in Conspiracy Theories.” Zeitschrift für Psychologie 232, no. 1: 7–17. 10.1027/2151-2604/a000545.

[sjop70111-bib-0046] Uscinski, J. E. , M. E. Adam , C. Klofstad , and J. Stoler . 2022. “Cause and Effect: On the Antecedents and Consequences of Conspiracy Theory Beliefs.” Current Opinion in Psychology 47: 101364. 10.1016/j.copsyc.2022.101364. 35728357 PMC9547178

[sjop70111-bib-0047] van Prooijen, J. W. 2017. “Why Education Predicts Decreased Belief in Conspiracy Theories.” Applied Cognitive Psychology 31, no. 1: 50–58. 10.1002/acp.3301. 28163371 PMC5248629

[sjop70111-bib-0048] van Prooijen, J. W. 2020. “An Existential Threat Model of Conspiracy Theories.” European Psychologist 25, no. 1: 16–25. 10.1027/1016-9040/a000381.

[sjop70111-bib-0049] van Prooijen, J. W. 2021. “Conspiracy Thinking: A Scapegoat Is Always Useful.” UNESCO Courier 2021, no. 2: 42–45. 10.18356/22202293-2021-2-13.

[sjop70111-bib-0050] van Prooijen, J. W. 2022. “Psychological Benefits of Believing Conspiracy Theories.” Current Opinion in Psychology 47: 101352. 10.1016/j.copsyc.2022.101352. 35644093

[sjop70111-bib-0051] van Prooijen, J. W. , and M. van Vugt . 2018. “Conspiracy Theories: Evolved Functions and Psychological Mechanisms.” Perspectives on Psychological Science 13, no. 6: 770–788. 10.1177/174569161877427. 30231213 PMC6238178

